# *Clostridium difficile* Surveillance Trends, Saxony, Germany

**DOI:** 10.3201/eid1404.071023

**Published:** 2008-04

**Authors:** Florian Burckhardt, Anett Friedrich, Dietmar Beier, Tim Eckmanns

**Affiliations:** * Robert Koch Institute, Berlin, Germany; † Landesuntersuchungsanstalt Sachsen, Chemnitz, Saxony, Germany

**Keywords:** Clostridium difficile, incidence, population surveillance, letter

**To the Editor:** Vonberg et al. (*1*) recently commented on the increase of *Clostridium difficile* seen in US hospitals by using discharge diagnoses and confirmed the observation from the United States (*2*) with hospital discharge data from Germany during 2000 through 2004. *C. difficile* ribotype 027 has recently been isolated in Germany (*3*). We further contribute to the assessment of *C. difficile* as an emerging threat by looking at population surveillance data.

*C. difficile* is not a federal notifiable disease in Germany, which limits our ability to analyze national surveillance trends. However, in 2002 the state of Saxony implemented additional mandatory surveillance of community- and hospital-acquired infectious enteritis caused by laboratory-confirmed *C. difficile*.

To check for an increase in notifications due to reporting bias of gastroenteric diseases, we compared the quarterly incidence data from 2002 through 2006 with data on *Salmonella* spp. infections (usually reported by local general practitioners) and rotavirus and norovirus infections (both usually reported by clinics). The potential problem of reporting bias for gastroenteric diseases has been addressed recently (*4*). Information about age and sex of *C. difficile* patients was available for 2006 only.

Quarterly incidences for *C. difficile* in Saxony were from 1.7–3.8 per 100,000 population in 2002 and 2003 and continued to increase to 14.8 cases per 100,000 population in 2006 (Figure). This constitutes a 6-fold increase of the yearly average of *C. difficile* incidence rates between 2002 and 2006. The third quarter of 2005 experienced a sharp drop that could not be explained retrospectively and might have resulted from transition to new procedures for data collection and management.

**Figure:**
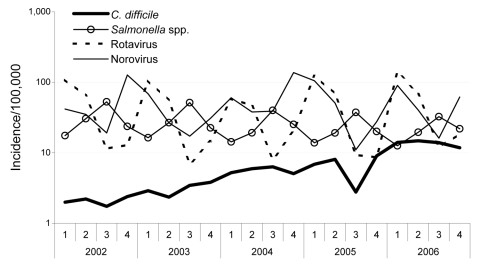
Quarterly incidence per 100,000 population of *Clostridium difficile* infections compared with gastroenteric infections caused by *Salmonella* spp., rotaviruses, and noroviruses in Saxony, Germany, 2002–2006. Note the log scale on the y axis.

Gastroenteric infections showed clear seasonality with a slightly decreasing yearly trend for *Salmonella* spp*.* and seasonal values from 13.8 cases per 100,000 in winter to summer peaks of 56.8. Rotavirus infections displayed an even stronger seasonality, with values from 7.0 cases per 100,000 in summer to winter peaks of 140.3. Norovirus infections peaked again during winter, at 137.2 cases per 100,000 but had as few as 11.0 cases per 100,000 during summer. Notification does not suggest reporting bias of gastroenteric infections.

Elderly persons, i.e., those >65 years of age, were affected most by *C. difficile* infections; this age group accounted for 1,506 (65%) of all cases (n = 2,306) in 2006. The 45- to 64-year age group had the next highest number of cases, 451 (20%). Men and women were affected equally in the different age groups; slightly more women (n = 805) than men (n = 701) with *C. difficile* infection were >65 years of age*.*

According to state and local health departments, there were no major health campaigns since 2004 that might have selectively increased awareness for *C. difficile* notification. Our results show a continuous increase of cases that even reaches seasonal notification levels of *Salmonella* spp. and Rotavirus infections, but the increase is difficult to explain entirely by changes in reporting behavior. We emphasize the role of individual German states in setting additional surveillance targets for public health. Given the epidemic potential and the severity of the disease, especially among the elderly, surveillance of *C. difficile* should be introduced throughout Germany along with enhanced prevention and treatment strategies (*5*).
